# Fast and Intelligent Irrigation System Based on WSN

**DOI:** 10.1155/2022/5086290

**Published:** 2022-07-14

**Authors:** G. Oussama, A. Rami, F. Tarek, Ahmed S. Alanazi, M. Abid

**Affiliations:** ^1^Department of Computer Science, Jouf University, Al Qurayyat, Saudi Arabia; ^2^CES-Lab, Sfax University, Sfax, Tunisia

## Abstract

In the agricultural industry, wireless sensor networks (WSNs) can be an important tool to promote economic growth. Using network devices in agriculture has the potential to enhance the production process. One of the key challenges WSN faces is energy efficiency. A model based on the water pipeline method is proposed in order to efficiently utilize sensor nodes in agricultural production and water distribution. A water pipeline serves as an important structure for transporting potable water across a distance for consumption or irrigation. In contrast, the biggest transportation problems of water pipelines are leaks. So, water resources may be lost as a result. These pipes need real-time monitoring to prevent such problems. The wireless sensor network technique, however, is considered one of the best solutions available today for monitoring water pipelines. A detailed analysis of agriculture is provided by the model. Aspects of WSN are discussed and their agricultural use is expounded. Moreover, this paper describes the various types of applicability of existing sensor networks in the field of agriculture, along with some technical perspectives. To achieve the best power consumption and communication for the two types of range, ZigBee wireless protocols are utilized. As such, high-performance information that provides a platform for WSNs to better support agricultural production is also included in our proposed model in order to address the shortcomings of existing WSNs regarding energy efficiency. This paper presents the improvements of the proposed solution compared to other techniques in the context of energy conservation in wireless sensor networks and in the monitoring water-saving process.

## 1. Introduction

Most countries around the world have always seen agriculture as an important industry in order to build their economies. A phenomenon such as farmland pollution, the degradation of the environment, and biodiversity destruction has been described as a result of excessive fertilization in agricultural production efforts [1]. Producing, growing, and developing the agriculture industry are heavily impacted by these factors. A good administration of agricultural production activities is essential to the sustainable development of modern agriculture. Both need to be taken into consideration in order to develop a scientific production technology. The application of WSN for energy efficiency can have a profound impact on agriculture. Management strategies for agriculture can greatly improve energy efficiency. Agricultural production is improved by utilizing information technology in a reasonable and continuous way. It also reduces pollution levels by using a variety of resources. Energy and water are part of the used resources. The production of agricultural products becomes more advanced and more accurate.

Human survival requires access to water. Approximately 96.6% of the water on Earth comes from the ocean. Of that, approximately 2.5% is fresh and can be used for agriculture. A vast amount of fresh water is transferred worldwide via millions of kilometers of pipelines at present.

**Figure 1:**
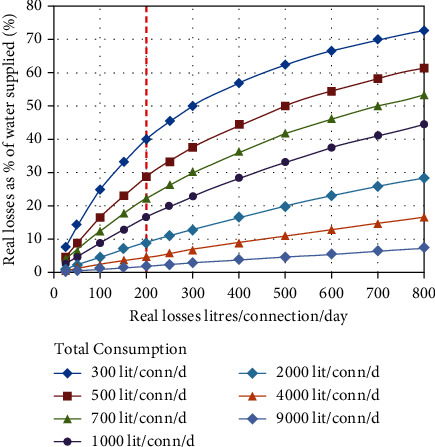
The NRW indicator of real water loss.

According to this work [2], a vast network of underground pipelines has been laid to connect Saudi Arabia's cities with the Arabian Gulf. A pipeline can be used for irrigation, making it easier for farmers to water their land. World Bank estimates that more than 45% of freshwater produced newly is lost in nonrevenue water (NRW) in third world countries. A recent study [3] reported the NRW indicator of real loss in terms of liters/connection/day in the world as described in Figure 1.

**Figure 2:**
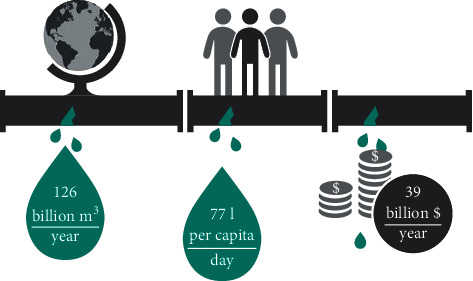
The wasted water around the globe.

It is expected that a pipeline with a large diameter will leak at least once per year, according to statistical analysis. Several environmental factors may cause damage to pipelines, including extreme soil conditions, aging of materials, bedding, and excessive loads. Excessive costs for hydraulic leaks can lead to infrastructure damage and environmental pollution. Thus, maintaining the pipeline infrastructure and ensuring its security is one of the main concerns. Based on Figure 2, the wasted water in the globe is calculated as 126 billion m^3^/per year, 77 liters per day with an annual amount of 39 billion dollars.

Traditionally, pipeline monitoring has been researched through architecture, sensing devices, and modules. For example, the authors of this work [4] assessed the impact of network architecture on network consistency. Recent studies and advanced works on this topic are reviewed in [5] by reviewing WSN technology applied to the supervision of water pipelines as well as underwater, aboveground, and underground pipelines. Also, in their study [6], they described and compared the characteristics of microcontrollers used to monitor water pipelines. Research in this work proves useful in helping to select the best existing systems and technologies, compared to work in other described works.

In Saudi Arabia, agricultural development has yet to reach its full potential and continues to face many challenges. The use of irrigation and fertilizers should be fully regulated and monitored using modern scientific methods. A proper monitoring and control process should also be in place [7, 8]. Agricultural research is still in its early stages, and there is a number of key technical challenges to overcome. Chemical fertilizers and irrigation, for example, can be used to make food production much better. A lack of standardization research has led to the product not reaching market scale because sensors are too diverse and expensive to be used in agriculture to overcome these challenges. Agricultural sensors have many limitations at present: poor irrigation techniques, poor working conditions, and short battery life. Additionally, since the farming production zone is located in a remote area, the public telecommunications network is inadequate and sometimes costly. Also, interference issues and spectrum compatibility make communication difficult. Agricultural applications employ WSNs for a range of activities, including measuring temperature, monitoring the environment, monitoring irrigation systems, and measuring water supply. Farmers can generate high-quality crops through the use of WSNs. To power sensor nodes, a battery power supply is required. So, as a result of these networks, crops are more expertly produced and the economy is benefited.

Several recent studies have found that the use of these networks will solve a lot of obstacles in agriculture implementation. This latter utilizing wireless sensor networks is a highly efficient method for improving crop yield, lowering farmer burdens, and improving productivity. It is important to use WSN for agriculture to ensure the healthy growth of crops [9, 10]. By using this method, weeds and pests can be effectively controlled and sustainable agriculture is achieved. With the use of the WSN, we can collect and sense all types of data in real time in the agricultural production process which will provide users with timely feedback. Users are informed of the results of data analysis and processing to ensure efficiency in agriculture management.

An analysis of communication protocol development potential in WSN for agricultural production is presented in this paper. This article focuses on the characteristics of WSNs and how they can be applied to agriculture. It outlines crucial factors that influence monitoring models. Additionally, it discusses some technical prospects that can be helpful in improving agriculture's overall development level effectively and comprehensively. Besides, this study explains how existing sensor networks are applied to agricultural production technology and their limitations. The organization parts of this paper are presented as follows. The state-of-the-art studies about our work, in Section 2, are described. Then, our proposed Smart Water Irrigation System (SWIS) is discussed in Section 3. Also, we demonstrate our monitoring of diseases in Section 4. Finally, simulation results are provided in Section 5 and followed by concluding remarks in Section 6.

## 2. Related Work

**Figure 3:**
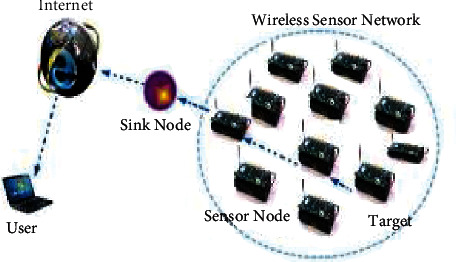
Wireless sensor networks architecture.

Battery-powered sensor nodes are used as an energy supply in the evolution of technology that is deploying rapidly in WSNs [11, 12]. This latter is formed by detached and dedicated sensors that observe, record, and transmit information about the surrounding environment to a central hub. A detailed representation of the WSN architecture and structure is given in Figure 3. The battery-powered sensor network includes RF modules, processors, and sensor nodes.

Communication is possible between the sensor node and the base station or coordinator node via a wireless communication link. Each node in a network collects, computes, and communicates data and information with the associated nodes. Information like humidity level, pressure, and temperature collected from sensor nodes can range from simple to complex. Then combined with each other, they achieve real-time monitoring in WSN. Accordingly, the sensor nodes monitor, store, and process data during the entire monitoring process [13]. Information will be sent to the WSN in order to perform real-time monitoring. The use of WSNs in farming has a wide range of benefits in terms of obtaining accurate and sufficient environmental information [14]. So, the sink node, base station, and sensor node are considered the three main components of WSN [15].

Nodes on the sensor network can communicate and compute. Wireless connections are made using short-range wireless technology, which can create multihop wireless networks.

It is possible to monitor fully the crop data to obtain general agricultural information and support agriculture development activities through the use of wireless sensor networks [16]. As wireless technology itself, WSNs are simple and of low cost and low power. It adapts to the agricultural production environment and is stable over time. Integrating modern agricultural characteristics into data acquisition algorithms can significantly increase data collection rates and bring on a better agricultural production. For better quality of service (QoS) and more efficient farming production, WSNs are being widely used in agriculture right now. The sensors are being used for collecting various information types in agriculture immediately (e.g., carbon dioxide levels, humidity, etc.). In addition to supporting agriculture and improving yields, the WSN can also be utilized for other agricultural applications. Using soil nutrient data, it is possible to predict crop health and product quality [17]. Also, you can use it to predict irrigation needs as well as soil moisture levels. The network can be expanded by adding relevant sensor nodes to improve parameter agricultural monitoring. Although WSNs have some applications in agriculture, some challenges remain, like deriving the optimal deployment scheme, communication range, and others [18]. It takes a long time to collect data from decentralized sensor nodes, and signal attenuation can weaken or destroy the communication link.

Power consumption is identified as a major problem in wireless sensor networks and, also, battery life extension because these nodes are powered by batteries, which have a limited amount of power. Even though WSN has been gaining popularity for several years, its application has some limitations [19]. For example, to eliminate the problem of sending data from farmland to the base station, a mobile data connection service is used. A base station located in a farmland region can communicate wireless sensor data to the sensor nodes, which enables the wireless sensor battery to be buffered. The power consumption of WSNs is extremely low. A design that has low power consumption, wide coverage, multiple connections, and low cost is adopted. WSN is extensively used in agricultural production to advance the quality of crops in the field of agriculture, where it plays an important role in monitoring the agriculture field [20]. With WSN technology rapidly developing, battery power is still a major challenge.

In another part, we have also investigated the concept of a wireless sensor network to detect leaks. A multimodal system was usually considered to detect leaks in most studies. Using humidity and temperature sensors, Sadeghioon et al. [21] detected leaks. Data was collected for a few days from the humidity sensor and the temperature sensor. Analyzing the pressure profile and temperature data, they concluded that there was a leak. Studying wireless sensor network standards was the focus of this work [21]. To determine where the leak is, they used five flow sensors. To find a leak and its location [22], the authors used a variety of sensors such as humidity, temperature, pressure, and a gas detection sensor in the same way as [23]. Furthermore, they demonstrated the wireless data transfer between Arduino and XBee. They calculated the optimal placement of flow sensors as seen in [24, 25]. Our system was built using these analyses. In the next section, we will discuss the power management used in irrigation when based on the use of water pipelines as well as different materials, monitoring techniques, data used, etc.

## 3. Different Monitoring Models

### 3.1. Sensors Types

A pipeline monitoring system is typically composed of three kinds of sensors, the most commonly used of which are as follows:

- First model: A static sensor is usually attached to the pipeline surface and reads the actual state of the pipeline [26].
- Second model Mobile sensor. The cells on this model travel from a source point to a sink point inside the pipe. Sensor data is copied from its memory to the backend system. Sensor observations, as well as location information, are provided in this information [27].
- Third model entitled Automotive Vehicles: Robots can either repair the damaged segment or gather information from it, but in most cases, static sensors capture information from pipelines, whereas robots or vehicles just collect the information.

### 3.2. Data Exploitation Types

Various methods of pipeline monitoring are used to carry out the monitoring process, and they include the following:

- The first method named centralized exploitation: the collected data is sent to the sink for processing, control, and alerting events when problems occur. Compared to other, a centralized algorithm is more accurate in providing location information. The aggregated data can also be analyzed to find the most interesting trends. However, its high bandwidth and power consumption make it expensive [28].
- The second method named distributed exploitation: it was designed to distribute the monitoring area into smaller sections in order to reduce the loading on nodes. Monitoring with this model, it is possible to locate faulty pipeline areas more quickly and begin the maintenance process right away. It aims to enhance the lifetime of the WSN by transmitting just the important data to a central unit. As a result, there are a lot of messages exchanged, which makes distributed exploitation energy-intensive [29].

### 3.3. Sensor's Coverage

Diverse sensor topologies and implementation methods are used. Monitoring pipeline length is the main objective of sensor deployment. We will examine the sensors' range and direction of communication in this section. There are two categories of sensors coverage, which are as follows:

- Horizontal coverage: some vehicles move at different angles, inside or outside of the pipeline, to improve monitoring coverage. They are known as autonomous underwater vehicles (AUVs). A vehicle collects information on its own or gathers sensor data in a horizontal fashion in order to send the information vertically to the subsequent sensors. This approach is used in some systems such as [30].
- Vertical coverage: it is achieved by implanting the motes along the monitoring pipeline, and they communicate linearly. Taking network operations centers into account, it can be found at the end of a pipeline or at both ends [31].

### 3.4. Power Management

Pipeline monitoring systems are sensitive to the power supply. Throughout the lifetime of a pipeline, which will last for many years, it needs to be monitored. In addition, the system must be durable.

Various methods have been developed for this proposal, including data filtering and aggregation, solar cells, and routing protocol management [32].

## 4. Monitoring Techniques for Water Pipeline

Over the years, methods of measuring total losses including actual and apparent leakages have been investigated. It is, therefore, necessary that new technologies are required to detect, locate, and estimate leak sizes.

### 4.1. Hardware-Based Methods

Through the use of specially designed accurate devices, these techniques can detect and locate leaks. Based on the nature of the detection equipment, hardware-based methods are categorized into five categories.

#### 4.1.1. Visual Inspection Technique

A leak can be detected this way traditionally. Personnel who are experienced in this technique are also required when inspecting prospective leaks. Using video, drones, and others to capture images of pipelines ground, visual inspections can reliably detect bursts and other problems along the pipelines. The software provides user-friendly real-time three-dimensional (3D) images [26]. Underground pipelines are not adequately supervised with this technique.

#### 4.1.2. Fiber Optic Sensing (FOS)

In order to provide information about the circumference of a pipeline, fiber optic cables are buried directly beneath the pipe and/or helically instrumented around it. Leak detection can be done using this method by analyzing the temperature change caused by water leaks [4, 32]. Due to its resistance to interference, this technique has the significant advantage of being unaffected by electromagnetic waves. Because the method requires an additional excavation for installation, it cannot be implemented in underground pipelines.

#### 4.1.3. Ground-Penetrating Radar (GPR)

The scanning of the ground surface is done using this technique. A buried object's size and shape can be determined with these drawings. It is possible to deploy some miles of ground-penetrating radar per day with ground-penetrating radar (GPR) [33]. So, investigation and time-consuming represent the costly drawbacks of this technique.

#### 4.1.4. Soil Properties (SP)

In order to detect leaking pipeline branches, the transported water such as well as soil temperature must be abnormal in value [34]. Explicitly uncovered pipelines without soil surrounding them cannot be treated with this method.

#### 4.1.5. Acoustic Detection (AD)

Excessive use of junctions in underground pipelines can result in leakage occurrences. The entire pipeline network is therefore usually monitored using an acoustic sensor. Its main benefit is the ability to detect damage indefinitely [24].

The disadvantages of this famous technique include its high cost, which necessitates the installation of many nodes in the monitoring area, which limits its application.

### 4.2. Software-Based Methods

By constantly monitoring various factors of the pipeline, like pressure, temperature, and so on, software-based techniques are primarily aimed at detecting and localizing leaks. The methods based on software can further be categorized into five groups.

- Negative Pressure Wave method (NPW): It is a method used for identifying leaks in a large space using stationary running strategies. Various parameters affect this technique including wave speed, noise from the device, and pressure changes.
- Mass Balance method (MB): It controls the difference between the upstream and downstream flow measurements within a specified interval to detect damage.
- Inverse Transient Analysis (ITA): A comparison is established between the signal reflected by monitoring devices and the signal observed in normal system operation.
- Pressure Point Analysis (PPA): Various pressure measurements need to be taken along the pipeline on a continuous basis. Detection of small leaks will be easy.
- Real-Time Transient Modeling (RTTM): It provides a fast rate of detection by continuously analyzing the pipe configuration and the product characteristics at the same time.

### 4.3. Monitoring Water Pipelines: Methods Evaluation

In Tables 1 and 2, we summarize the key features of spoiled detection systems for every technique so that we can compare their performance. According to the studies referred to previously, these results are based on confident research. In Table 1, the utility of implementation and positioning of sensors are presented for each technique. Furthermore, in Table 2, the techniques' accuracy is also evaluated by calculating the percent of damaged objects identified perfectly. In addition to accuracy, there are four other key characteristics to assess. The first one is sensibility, which represents the capacity to detect small leaks. The second one is named the availability, which means the ability to conduct continuous monitoring 24 hours a day. The third type is the false alarm. This latter involves fewer false alarms in a used good technique. Finally, detection speed is defined as high when the detection time is up to a few minutes. It is then classified as medium when it is discovered within a few hours and as low for longer periods.

The implementation characteristics are also compared in Table 3, considering factors such as cost, retrofitting, complexity, and maintenance. The supported function is mentioned by (+), and the not supported function is mentioned by (/).

All of these tree tables mention that for all the key factors, tools that are rated “good” cannot be found. So, hardware-based models, ground-penetrating radar, and optical fibers cannot be used because of their limitations and they are expensive. Also, software-based techniques cannot be used because they cannot estimate the leak location perfectly and are unreliable in detecting damages [22]. The progress in recent years has been considerable; however, there is still much scope for improvement, especially in terms of real-time modeling and detecting pipe damages. In essence, the purpose is to provide a system of heterogeneous wireless sensor nodes that can detect leaks in water pipelines and monitor them with different nodes of different architectures. High safety levels, high localization precision, and real-time detection are all characteristics of these technologies.

## 5. Water Pipeline Monitoring System Based on WSNs

According to the communications technology used, monitoring water pipelines can be categorized into several categories. So, supervisory techniques include manual techniques, wired systems, SCADA systems, and wireless sensor networks.

Table 3 outlines a general assessment of each technique. As an additional comparison, Table 4 presents some of their key features. It takes into account a variety of factors, including flexibility, autonomy, security, and cost. In the table, the (/) indicates that there is not enough information to draw any conclusions.

Manual networks and WSNs both demonstrate their versatility with regard to extending or deleting models without network problems in Tables 3 and 4. Then, SCADA and wired networks show low levels of flexibility. Moreover, SCADA and wired systems are very expensive to install and maintain, while manual inspection is expensive, but WSN systems are even cheaper. The application based on WSNs is more accurate, of lower cost, and easy to set up than traditional methods.

### 5.1. Wireless Sensor Networks (WSNs)

Science and industry have become more interested in wireless sensor networks. Wireless development, microelectronics, energy conservation, and microelectromechanical systems (MEMSs) have all been impacted by the development of this innovation over the last few years. There are many sensor nodes in a wireless sensor network, which are constantly monitored. A wireless connection is used to connect these wirelessly embedded nodes. Their joint effort collects large quantities of accurate information about many locations. Data is then processed and sent to sink points, also called gateway nodes. Their efficiency has been demonstrated in a variety of domains. An information structure transforms physical sensor information into general electronic measurements that can then be exploited by the wireless sensor node.

Sensor nodes consist of a variety of units, such as sensing, processing, and communication. In a measurement network, in general, the power consumption is managed via batteries, which is an important constraint. Moreover, it is advisable to implement sensors in an elegant manner to reduce communication costs and extend the lifespan of the network.

### 5.2. Current Work

Monitoring systems have been implemented using WSNs in several projects. Our analysis summarizes the characteristics of damage detection systems according to each of the different techniques in order to compare their performance. Data exploitation and placement pipeline depend on sensor coverage, parameters, and sensor types. Pipeline monitoring applications implemented in existing systems use a centralized monitoring system like [39] or [21] in which a powerful node is present. A large number of sensors used in this type of system create scalability problems. Some projects, such as [22], implemented a distributed monitoring system which was more appropriate for monitoring a large pipeline network. They made use of mobile sensors to overcome the scalability issue. In a highly dynamic network, the number of messages and energy used by this solution will be expensive.

**Figure 4:**
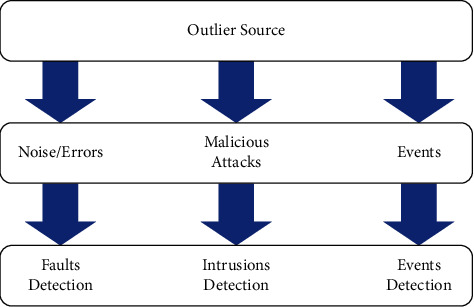
Different types of outlier sources in wireless sensor networks.

WSNs-based systems present some challenges and limitations as they require protocol management as well as tool qualification. Modern algorithms are needed for outlier detection, energy conservation, security management, and self-location of targets to fulfill these purposes. The goal of outlier detection methods is to cleanse the gathered data and build the most helpful information for end-users, so they can make the most informed decisions. The source of outlier data is divided into three categories: event, malicious attacks, and noise. The outlier data detected in water pipelines may be due to damage [40]. This latter can cause more abnormal data to be detected. The purpose of outliers is to identify sudden changes in pressure profiles caused by noise, events, or malicious attacks, as shown in Figure 4.

Additionally, the purpose of these approaches is to conserve energy while preserving low communication exchanges so that a decision can be made accurately and efficiently. The tools are also designed to detect and localize damages in a water network, ensuring continuous monitoring of the system [41].

## 6. LEACH Algorithm

### 6.1. Network Architecture

**Figure 5:**
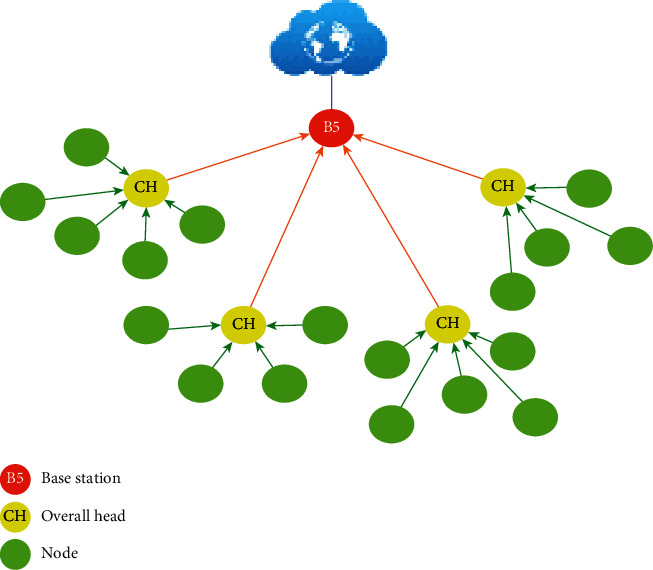
Classification method based on WSNs.

LEACH is a protocol that is based on the concept of classification (clustering) shown in Figure 5, which consists of partitioning the network into groups (clusters). The nodes send their data to the CHs which in turn send this data to the BS. The CHs perform simple processing (e.g., aggregation) on the data before transmitting it.

### 6.2. LEACH Protocol

The operation of the LEACH protocol is as follows: the nodes self-form to be CHs. They are based on the desired percentage of CH in the network and on the number of iterations during which a node has taken the role of CH. A node “n” takes a value between 0 and 1; if this value is less than a threshold *T*(*n*), the node declares itself CH.

(1) $$\begin{array}{c} T\left(n\right)=\frac{K}{N-K\text{ mod}\left(r,N/K\right)}\text{if }n\in G,\\ T\left(n\right)=0\text{ if not,} \end{array}$$

where *k* is the desired percentage of CHs, *r* is the current iteration, and *G* is the set of nodes that have not been selected as CHs during the last (*N*/*K*) iterations. Then, each CH informs its neighbors of its election, and each node chooses the closest CH.

## 7. Existing Upgrades to Extend Lifetime

The objective was to favor the nodes which have more energy to become CH. To achieve this, the authors use the formula which makes it possible to calculate the ratio of energy present in the node (*E*_pres_) on the initial energy (*E*_ini_) and introduce it into the classification formula, the new threshold considering the energy below:

(2) $$\begin{array}{c} T_{1}\left(n\right)=T\left(n\right)\frac{E_{\text{pres}}}{E_{\text{ini}}}. \end{array}$$

## 8. Proposed Improvements

The goal of our solution is to favor the nodes which have more energy and are close to the BS to become CHs. So, we introduce the metric of the distance between the node and the base station.

### 8.1. Using Distance between the Node and the Base Station in the Selection Formula

The objective of this method is to give more chances to the nodes which are close to the base station so that they become CHs. So, the threshold equation becomes

(3) $$\begin{array}{c} T_{2}\left(n\right)=T\left(n\right)\frac{d_{\text{max}}}{d}, \end{array}$$

where (*d*) is the distance between the node and the base station and (*d*_max_) is the maximum distance between the base station and a node.

As we have already presented previously that the nodes self-form and randomly choose a number between 0 and 1 and this value must be lower than a threshold for the nodes to be CHs, so, this threshold must have a greater or lesser value so that we will have a high probability of having CHs nodes. If we take two nodes (*n*1) and (*n*2) which, respectively, have distances *d*1 *<* *d*2, we will trivially have *T*2 (*n*1) > *T*2 (*n*2).

### 8.2. New Selection Formula: Energy and Distance

This formula consists in considering the nodes having more energy and less distance compared to the base station as CHs; the idea is to make a combination between the two formulas *T*2 and *T*1 with such percentages (*a*) and (*b*). After some changes to this latter, we got the following result:

(4) $$\begin{array}{c} T_{4}\left(n\right)=aT_{2}\left(n\right)+bT_{1}\left(n\right), \end{array}$$

avec *a*=0.15 and *b*=0.85(*a*+*b*=1).

### 8.3. Experimentation and Results

**Figure 6:**
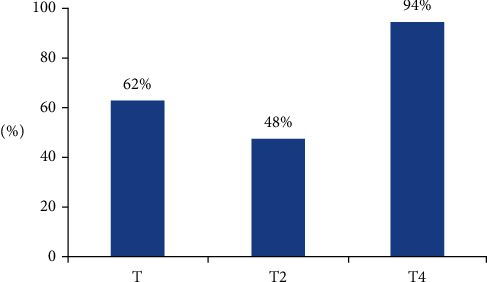
Network lifetime percentage of our proposed method compared to original leach.

To evaluate the performance of the LEACH protocol, we based ourselves on the existing improvements as well as on the improvement that we have proposed. We used the NS2 simulator (Network Simulator). The simulation results exhibited in Figure 6 clearly show that there is an extension of the network lifetime, applying the new formula T4.

Figure 6 shows that the T4 formula keeps all nodes together alive for 94% which reflects a further extension in network lifetime. On the other hand, by applying the original LEACH formula, the nodes could only resist 62%. However, we managed to extend the lifetime of the network by 32%. This is an interesting result since, in wireless sensor networks, all the nodes must collaborate to accomplish a well-defined objective.

## 9. Development of Smart Water Irrigation System with Moisture Monitoring

According to the proposal irrigation system for water-saving plus moisture, monitoring is described in this section. The proposed system was developed taking into account the extremely hot climate of Saudi Arabia. Hence, the vast majority of the country is covered by the Sahara Desert. In our work, we have developed a water-saving irrigation control system that is based on wireless sensor networks [37], whereby the system comprises low-power wireless sensor nodes that communicate through an ad hoc ZigBee network. We will monitor soil moisture information parameters such as soil water degree, temperature, and relative humidity that can all be used to measure moisture potential. A four-channel temperature and humidity transmitter will be used to collect this data. The information details are determined by using the various component of the sensor. In the following subsections, we will describe in detail the proposed design [42].

### 9.1. Smart Water Irrigation System for Farmers

**Figure 7:**
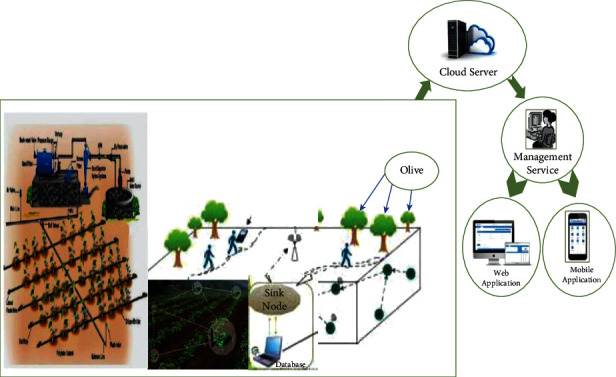
Smart Water Irrigation System (SWIS) based on wireless sensor networks.

As shown in Figure 7, our system of agriculture irrigation is based on famous technology entitled WSN which is made up of four components: an irrigation controller, receiving sensors, a set of sensors, and a network of irrigation pipes. To construct an irrigation node group, sensor nodes that bear soil moisture are disseminated in accordance with the planting and irrigation status of farming. Each node is in charge of keeping track of the soil moisture in a certain area [43].

A standard WSN, utilizing ZigBee technology based on transmitted wireless data, consists of an irrigation region and receiving sensors. Wireless multihop is used to transfer sensor data to the receiving node. Our discipline is designed to install a network for an irrigation pipe in the farming area in the irrigation region and an electrical control valve on the pipe to create an automated water-saving irrigation system. If the control of smart water irrigation is adaptable, the total system will be more versatile [44].

A water-saving irrigation system may be modified based on the original irrigation pipe network. For greater deployment of irrigation system pipe network and to save money, an electronic control valve can be fitted. The irrigation controller in the WSN coverage region may spray irrigation in specified locations based on sensor data. This system contains a specific module taking charge of network supervision. The proposed Smart Water Irrigation System is mainly dependent on wireless sensor networks and water pipelines [45].

### 9.2. System Hardware Structure

**Figure 8:**
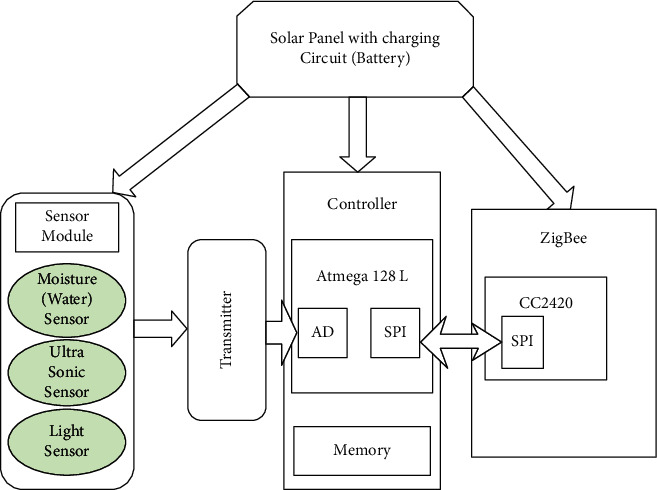
Hardware structure of the sensor node used in the proposed architecture.

In this part, the hardware structure of the sensor node implemented in the proposed architecture is addressed and illustrated in Figure 8. The controller module, sensor module, ZigBee protocol communication module, and solar self-powered module make up the majority of the hardware structure [46].

The irrigation controller is built using an embedded system development board as the mainboard. The receiving node receives information through a serial connection and processes the control data. The system is very scalable. The WSNs measure humidity which is realized 5 times in a minute (on a 12-second cycle) and send the data to the irrigation controller. When the irrigation controller detects that the humidity sensed by the WSN nodes in a specific location is lower than the prescribed value, it activates the irrigation network's electric control valve. The system will start irrigation and close the electric control valve of the pipe network in this region when the soil humidity in the area reaches a particular level. This hardware structure presents a very effective impact of improvements of the proposed solution compared to other techniques in the context of energy conservation in wireless sensor networks and in the water-saving process. It both minimizes energy consumption and wasted water [47].

## 10. Data Collection Methods for Prevention Diseases

An early warning system takes into account the Saudi Arabian country's environment. The data used in our work are humidity, pressure, and temperature. It can be provided by normal events, nose, or malicious attacks. Also, it is divided into data acquisition, data transmission, data processing, and data application. Diseases are a threat to plants of all kinds, but olives are most susceptible to pests because it affects the quality of the oil.

**Figure 9:**
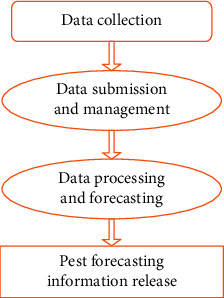
A diagram information chain and its main components.

A series of information is formed through the collection of data, data management, data processing, and forecasting, and finally, the forecasting information is released for monitoring and early warning. A diagram presented in Figure 9 is attached showing the information chain and its main components:

The technique of data gathering, transmission, processing, and application in information technology corresponds to each link of the information chain. In this section, we will discuss monitoring and early warning technologies. Sensor technology, database technology, expert systems technology, global positioning systems (GPS), geographic information systems (GISs), network technologies, and communication technology are all components of the disease monitoring and early warning information chain. For manual analysis of conventional diseases, the use of pad and GPS data acquisition and recording technology is most widely used. Data, as well as GPS positioning information, are simultaneously recorded. During the same time, data on field microclimates are collected, which are closely related to diseases. To obtain real-time microclimate data automatically, the field microclimate data monitoring technology utilizes sensors and communication technologies such as GPRS. Diseases are represented by the data, which is then transmitted to the database for analysis.

In another part, a data collection technology combined with data transmission technology forms the key components of the proposed system. Data management systems can benefit from real time, reliable monitoring data on diseases, and microclimates. This system takes into account environmental factors when it develops the expert system. This latter inference engine produces forecasts of diseases, which are then released through a forecast information release system in order to guide plant protection efforts [42, 43, 45].

## 11. Simulation of Agriculture Effects

For the agriculture effect, simulation analyses are performed here. A variety of physiological and ecological indicators of plant growth are necessary in order to monitor the growth status of plants and improve their scientific management level. Plant growth control activities were adversely affected by the outdated monitoring methods of the past. The use of WSNs in agriculture production is currently being actively introduced, which can study the state of crop growth automatically at a lower cost. The physiological and ecological parameters of plants are also monitored, analyzed, and recorded by an intelligent system. Accordingly, Figure 7 demonstrates how this latter can significantly improve the efficiency of monitoring.

**Figure 10:**
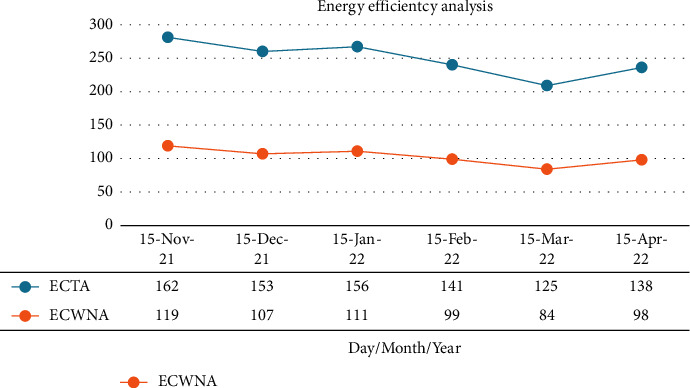
Energy consumption based on WSNs.

Economic growth has been hindered by a lack of water resources, but agricultural water is being wasted in large amounts. Irrigation research must be intensified to reduce water waste. Monitoring irrigation water and soil moisture during crop growth is a good application of the wireless network agriculture technology. Essentially, this latter system is a simple wireless sensor network (WSN) that allows for agriculture applications. Additionally, an irrigation scheme is developed in conjunction with the development of these crops. There is however little evidence of WSNs being applied to agricultural production, which mostly takes place in smaller production environments such as tea gardens, orchards, and greenhouses, and more research is required. In order to increase production, large-scale farmland is needed. With the increase of time, agricultural irrigation based on wireless sensor networks has played a very important role in water saving. To understand the growth status of plants and improve the science of management, it is important to monitor their physiological and ecological indicators. Plants' growth control activities were affected by the old-fashioned monitoring methods. Currently, WSN is actively utilized in agriculture production to sense crop growth at a lower cost while automatically sensing it. In addition to tracking, analyzing, and recording the physiological and ecological characteristics of plants, intelligent systems serve as a good premise for improving the efficiency of monitoring. Based on data obtained by sensors like temperature, humidity, and pressure, we analyze the state of the land and the consumed energy. A study of data between November 2021 and April 2022 shows that WSN-based agriculture has considerably lower energy consumption than traditional agriculture (mentioned in Figure 10).

## 12. Conclusion

One of the most challenging aspects of wireless sensor networks is their energy efficiency. Based on the water pipeline, this paper proposes an efficient way of utilizing the energy of wireless sensor networks for agriculture production. We are particularly interested in various monitoring techniques suitable for preserving the number of water pipelines as part of our work. To provide WSN with an information platform that can support a better role in agricultural production, the proposed model aims to improve on the shortcomings of existing WSN in the context of energy efficiency. Agriculture is a key factor impacting the global economy; therefore, WSN is important.

In the end, based on our solution, we conclude that 19% of water could be saved using this irrigation practice. Also, wireless sensor networks can improve accuracy and efficiency to achieve agricultural goals as well as reduce costs associated with wireless protocol systems. Agriculture can improve its management and production efficiency as a result of using an agricultural automation system.

## Figures and Tables

**Table 1:** Architectural damage monitoring methods evaluation.

	Methods	Detection	Localization	Sensors positions	
				Inside	Outside
Hardware	VI	+	+	—	+
	FOS	+	+	—	+
	GBR	+	+	—	+
	SP	+	+	—	+
	AD	+	+	+	—

Software	NPW	+	+	+	—
	MB	+	—	+	—
	ITA	+	+	+	—
	PPA	+	—	+	—
	RTTM	+	+	+	—

**Table 2:** Damage monitoring techniques evaluation.

	Methods	Cost	Accuracy	Sensibility	Size estimation	Availability
Hardware	VI	H	H	+	—	—
	FOS	H	H	+	—	—
	GBR	L	L	+	—	—
	SP	L	L	+	—	—
	AD	M	H	+	—	+

Software	NPW	L	M	+	+	+
	MB	L	L	—	+	+
	ITA	L	L	—	—	+
	PPA	L	H	+	+	+
	RTTM	M	H	—	+	—

NB: H = high, M = medium, L = low.

**Table 3:** General assessment of different techniques.

	Advantages	Disadvantages
Manual technique Wu et al. [27] Jawhar et al. [23]	Need a real-time information	Not applicable to underwater applications
		Complex & costly

Wired technique Wong et al. [35]	Faster & secure compared to a wireless network	Rapidly technique failure
		Hard access to pipeline region

SCADA Cheng et al. [30] Enache et al. [36]	Rapid installation	Lower sense ranging
	Adaptable for application with short range	Single point of system failure

WSNs Kartakis et al. [37] Cai et al. [38]	Portable & scalable	High conception in energy
	Low corrosion	Sensitive & not reliable

**Table 4:** Monitoring techniques evaluation-based communication.

	Used by:	Cost	Security	Flexibility	Autonomy	Complexity
Manual technique	Wu et al. [27]	M	L	H	L	—
	Jawhar et al. [23]					
Wired technique	Wong et al. [35]	H	M	L	M	M
SCADA	Cheng et al. [30]	H	H	L	H	H
	Enache et al. [36]					
WSNs	Kartakis et al. [37]	L	H	H	H	—
	Cai et al. [38]					

NB: H = high, M = medium, L = low.

## Data Availability

The data used to support the findings of this study are included within the article.
